# Altered Cortical Swallowing Processing in Patients with Functional Dysphagia: A Preliminary Study

**DOI:** 10.1371/journal.pone.0089665

**Published:** 2014-02-19

**Authors:** Sonja Suntrup, Inga Teismann, Andreas Wollbrink, Tobias Warnecke, Martin Winkels, Christo Pantev, Rainer Dziewas

**Affiliations:** 1 Institute for Biomagnetism and Biosignalanalysis, University of Muenster, Muenster, Germany; 2 Department of Neurology, University of Muenster, Muenster, Germany; Julius-Maximilians-Universität Würzburg, Germany

## Abstract

**Objective:**

Current neuroimaging research on functional disturbances provides growing evidence for objective neuronal correlates of allegedly psychogenic symptoms, thereby shifting the disease concept from a psychological towards a neurobiological model. Functional dysphagia is such a rare condition, whose pathogenetic mechanism is largely unknown. In the absence of any organic reason for a patient's persistent swallowing complaints, sensorimotor processing abnormalities involving central neural pathways constitute a potential etiology.

**Methods:**

In this pilot study we measured cortical swallow-related activation in 5 patients diagnosed with functional dysphagia and a matched group of healthy subjects applying magnetoencephalography. Source localization of cortical activation was done with synthetic aperture magnetometry. To test for significant differences in cortical swallowing processing between groups, a non-parametric permutation test was afterwards performed on individual source localization maps.

**Results:**

Swallowing task performance was comparable between groups. In relation to control subjects, in whom activation was symmetrically distributed in rostro-medial parts of the sensorimotor cortices of both hemispheres, patients showed prominent activation of the right insula, dorsolateral prefrontal cortex and lateral premotor, motor as well as inferolateral parietal cortex. Furthermore, activation was markedly reduced in the left medial primary sensory cortex as well as right medial sensorimotor cortex and adjacent supplementary motor area (p<0.01).

**Conclusions:**

Functional dysphagia - a condition with assumed normal brain function - seems to be associated with distinctive changes of the swallow-related cortical activation pattern. Alterations may reflect exaggerated activation of a widely distributed vigilance, self-monitoring and salience rating network that interferes with down-stream deglutition sensorimotor control.

## Introduction

Functional swallowing disorders including functional oropharyngeal dysphagia, globus pharyngis, phagophobia and heartburn have not been well studied. The term “functional symptom” describes a distortion of a body function that cannot be explained by an underlying pathophysiological disease mechanism or structural lesion. According to current psychological theories functional symptoms can be attributed to stress and emotional disturbances. However, rather than using the term “psychogenic”, speaking of “functional” symptoms is more acceptable to patients and better reflects rare scientific knowledge about these conditions [1]. A literature search in PubMed using the terms “psychogenic” AND “dysphagia” returns only 36 entries. Already in 1989, Ravich et al. concluded that the strong resentments against this diagnosis have hindered its scientific investigation - contrary to its frequent use in clinical practice [2]. Functional dysphagia is currently regarded as a legitimate diagnosis in single cases, in which thorough evaluation of the oral cavity, pharynx and esophagus with sophisticated diagnostic methods fails to reveal any organic reason for the patient's complaint [3], [4]. According to Galmiche et al. (2006) [5] diagnostic criteria for functional esophageal disorders must include all of the following: (1) Sense of solid and/or liquid foods sticking, lodging, or passing abnormally through the esophagus; (2) absence of evidence that gastroesophageal reflux causes the symptom and (3) absence of esophageal motility disorders. These criteria must be fulfilled for three months, with symptom onset at least six months before the diagnosis is made. For functional oropharyngeal dysphagia, similar criteria can be applied, except that functional symptom perception is located in the oral cavity or pharynx instead of the esophagus.

Psychological dysfunction has been considered as a contributing factor for functional dysphagia. Psychogenic dysphagia patients have been reported to show clinically significant levels of psychological distress and particularly anxiety [6]. In another study, however, a battery of standardized psychological tests for somatization, depression, and anxiety failed to distinguish between patients with functional dysphagia and those with dysphagia due to organic causes [7]. Hence, the etiological significance of these psychological factors remains uncertain.

Recent research applying advanced functional neuroimaging techniques provides a growing body of evidence for objective neuronal correlates of functional symptoms, thereby shifting the disease concept from a psychological towards a neurobiological model [1], [8]. Sensorimotor abnormalities involving peripheral or central neural pathways have also been suggested as underlying pathogenetic mechanism of functional dysphagia [5], but to the best of our knowledge this interesting hypothesis has not been explored so far. In the present preliminary study we therefore investigated cortical swallow-related activation in patients diagnosed with functional dysphagia by means of magnetoencephalography (MEG) to determine, whether functional dysphagia is associated with alterations in cortical swallowing processing. The results are discussed in the context of current theories and literature on functional symptoms with a focus on neurogastroenterology.

## Methods

### Participants

Over a 3-year period ten patients have been diagnosed with functional dysphagia from the neurological dysphagia outpatient clinic at our university hospital. In all cases, extensive examination (including fiberoptic endoscopic evaluation of swallowing, videofluoroscopy, gastroscopy, esophageal and pharyngeal manometry, X-ray of head and neck, diagnostic cerebral MRI scan, cerebrospinal fluid examination, investigation for myopathy, motor neuron disease or neurodegenerative disorder where appropriate, depending on the patients clinical presentation) had revealed no organic origin for the subjective complaint of a feeling of foods (mostly solid food, but occasionally also liquids) sticking in the throat or passing abnormally through the pharynx during meals. Five of the ten patients were included in this study. Four subjects had known psychiatric comorbidities and/or were taking medication affecting the central nervous system and were therefore not eligible to take part because this might have influenced brain activation pattern. One of the eligible patients refused to take part.

Besides the above mentioned complaints, three study participants also described a globus sensation. One patient felt difficulty initiating a swallow, which occasionally even led to phagophobia. Although symptom duration ranged between 2 and 4 years relevant loss of weight was negated by all patients. They were not diagnosed with any other eating disorder, neurological or gastroenterological diseases. None of the patients took any prokinetic drugs.

Five healthy age- and sex-matched volunteer control subjects (see table 1) recruited from the local population without any medication, history of dysphagia, or any psychiatric, neurological, gastroenterological or ear-nose-throat disorder served as controls.

**Table 1:** Subject characteristics and behavioral parameters (mean ± SD).

	Healthy subjects	Patients	p-value
**Subjects (n)**	5	5	
**Sex (female/male)**	2/3	2/3	
**Age (years)**	40.2±16.1	41.8±12.5	0.865
**Head movement during MEG (mm)**	4.7±3.3	5.7±2.9	0.685
**Swallow count during MEG (n)**	53±9	50±24	0.773
**Swallow duration M0–M2 (s)**	2.1±0.9	2.9±0.9	0.220
**Swallow duration M0–M1 (s)**	0,89±0.4	1.40±0.4	0.094
**Swallow duration M1–M2 (s)**	1.24±0.5	1.50±0.6	0.496
**EMG power (µV)**	83.0±44.9	63.7±54.4	0.558
**EMG amplitude (µV)**	423.3±139.5	398.6±204.1	0.769

### Ethics statement

The local ethics committee at the Medical Faculty of the University of Muenster approved the protocol of the study. Written informed consent was obtained from each subject after the nature of the study was explained, in accordance to the principles of the Declaration of Helsinki.

### Data acquisition

MEG data were collected using a whole head 275-channel SQUID sensor array (Omega 275, CTF Systems Inc.). During the MEG measurement of 15 minutes duration, each subject swallowed self-paced without external cueing. To facilitate volitional swallowing water was infused into the oral cavity via a flexible plastic tube 4.7 mm in diameter attached to a fluid reservoir. The reservoir bag was positioned about 1 m above the mouth of each subject when seated. The tip of the tube was randomly placed in the left or right corner of the mouth between the buccal part of the teeth and the cheek and gently fixed to the skin with tape. The infusion flow was individually adjusted to the subject's request and ranged between 8 and 12 ml/min. Swallowing acts were identified by surface electromyographic (EMG) recording with bipolar skin electrodes (Ag-AgCl) placed on the submental muscle groups [9]. The electrodes were connected to a bipolar amplifier (DSQ 2017E EOG/EMG system, CTF Systems Inc., Canada). Magnetic fields were recorded with a sample frequency of 600 Hz. During acquisition the data were filtered using a 150 Hz low-pass filter. The participants' head movements were continuously recorded and the number of swallows performed during the measurement session was counted for each subject.

### Data analysis

Statistical analyses on subject characteristics, swallow count, head movement and electromyographic parameters collected during the MEG measurement (see below) were carried out using SPSS 20.0 (IBM Corp., USA). The assumptions of a normal distribution and equality of variance in the data were confirmed in all variables using the Kolmogorov-Smirnov-Test as well as Levene's test prior to any further analysis. For comparison between subject groups independent-sample t-tests were applied.

**Figure 1:**
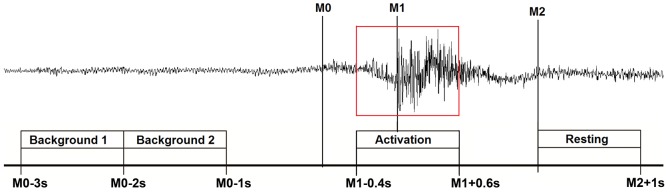
Definition of movement and resting stage according to swallow-related submental muscle activity. The surface EMG trace of a single swallowing act is shown.

MEG Data analysis was performed as previously published [10]–[12]. In brief, for event-related analysis of the MEG recordings each individual's EMG signal was used to mark the beginning of main muscle activation (M1) and the end of the task-specific muscle activity (M2) for every single swallow (see figure 1). The beginning of the main muscle activation was defined as an enduring >100% increase in amplitude or frequency of the EMG signal after an initial increase of more than 50% of EMG activity defining the onset of swallowing preparation. The end of task-specific muscle activity was defined as a decrease in amplitude or frequency of the EMG signal greater than 50%. To estimate the maximal null distribution (see below), a third marker was set to distinguish background activity from the onset of swallowing preparation (M0). EMG data were baseline-corrected and high pass filtered with 0.1 Hz before markers were manually set. The examiner who set the markers to the datasets was blinded to the subjects study group allocation. For further analysis time intervals were defined as following:

1. Active stage: −0.4 to 0.6 s in reference to M1
2. Resting stage: 0 to 1 s in reference to M2
3. Background stage 1: −3 to −2 s in reference to M0
4. Background stage 2: −2 to −1 s in reference to M0

To compare swallowing behavior during MEG data acquisition the mean power (root-mean-square value) and peak-to-peak amplitude of the submental EMG recordings were calculated across the active swallow stage (1) in all subjects and statistically compared as described above.

Synthetic aperture magnetometry (SAM), a minimum-variance beamformer technique, was applied for neuronal source localization. This method is capable of analyzing induced brain activity such as event-related desynchronization (ERD) of cortical rhythms which occurs during motor tasks [13], [14]. MEG also detects fields associated with tongue movement during swallowing since the tongue behaves like a current dipole. The use of SAM however, overcame the limitations of traditional dipole source analysis as described by Loose et al. (2001) [15] because the technique requires no a priori estimates of numbers or approximate locations of sources and can separately localize distinct sources that are active at the same time. While the artifacts caused by oropharyngeal muscle activation during the act of swallowing make it difficult to study activation in subcortical and brain stem structures, the cortical areas can be examined in detail. SAM has proved to be a reliable approach to examine the complex function of swallowing in humans [10]–[12], [16], [17]. MEG data were filtered within five different frequency bands: theta (4–8 Hz), alpha (8–13 Hz), beta (13–30 Hz), low gamma, (30–60 Hz), high gamma (60–80 Hz). From the filtered MEG data, SAM was used to generate 20×20×14 cm volumetric pseudo-t images [18], with 3-mm voxel resolution. A pseudo-t value cancels the common-mode brain activity by subtracting the source power found in a defined control stage from the source power in the active stage. To account for uncorrelated sensor noise, this difference is normalized by the mapped noise power [18]. For analyzing cortical activity during the active (1), the corresponding resting stage (2) served as control. The required similarity between the resting stage and the two background stages in patients as well as in controls was proven before by a direct comparison of these three stages. For analysis of single conditions, the significance of activated brain regions within each study group was assessed by the permutation test method described by Chau et al. (2004) [19]. The maximal null distribution was estimated here by comparing background stages 1 and 2 [19], [20]. For the comparison between groups, a standard permutation test for unpaired samples was performed [20]. Transformation into a common anatomical space is required for these analysis steps. Because the patients' diagnostic MRI scans did not fulfill the requirements for creating a scientific head model due to differences in image format and lack of coregistered fiducial markers, normalization was performed as previously published by our group [21].

Hemispheric lateralization of swallow-related activation was quantified using a lateralization index (LI), which was calculated as (L−R)/(L+R), where L and R is the cumulative pseudo-t-activation of the left and right hemisphere, respectively. A positive LI indicates left hemispheric lateralization, whereas a negative LI indicates stronger right hemispheric activation. A LI of about 0 represents indeterminate dominance, 1 and −1 indicate unilateral activation.

## Results

### Clinical data

The participants' demographic characteristics and behavioral data are presented in table 1. During the MEG measurement patients performed equally well to healthy subjects. Head movement and swallow count did not differ between groups. Coughing, choking or any other signs of swallowing dysfunction did not occur. EMG swallow parameters were comparable between groups. Total swallow duration (M0-M2) and the duration M1–M2 did not show significant differences between groups. However, there was a trend towards longer duration of the preparatory oral swallow phase (M0–M1) in psychogenic dysphagia patients (p<0.1).

### MEG results

**Figure 2:**
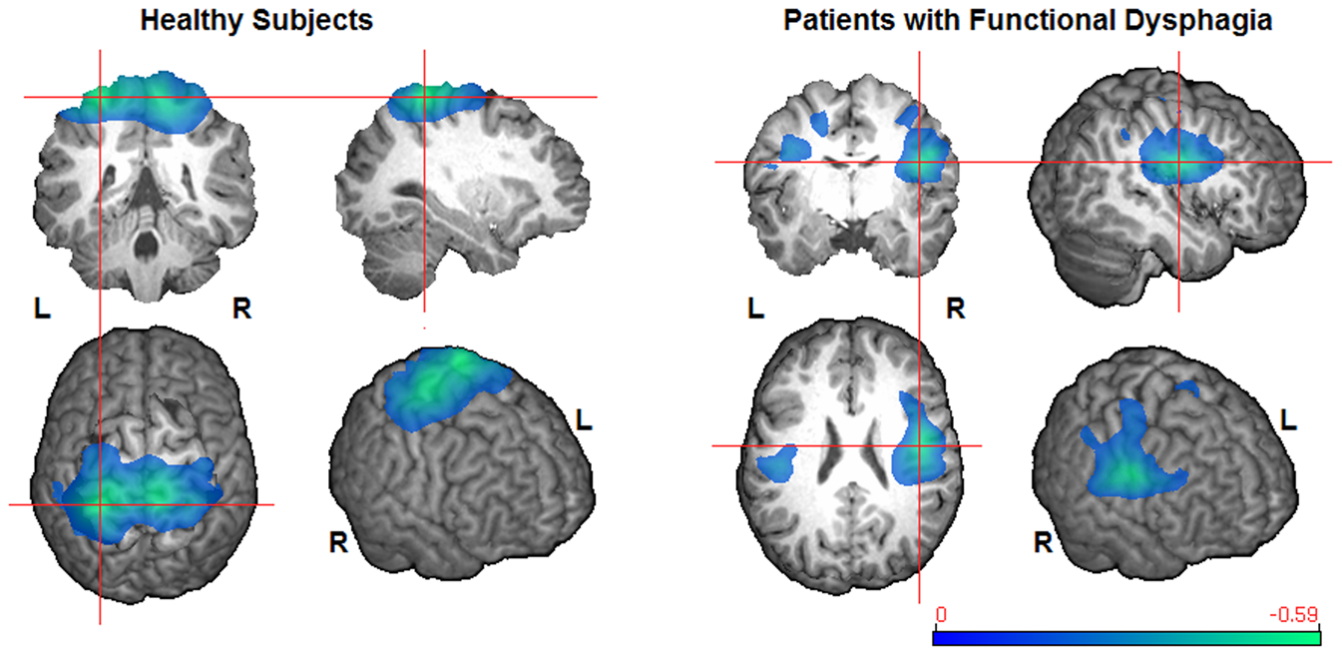
Group results of significant cerebral ERD during swallowing (13–30 Hz, p<0.05). The color bar represents the pseudo-t value; the crosshair indicates absolute peak location.

**Figure 3:**
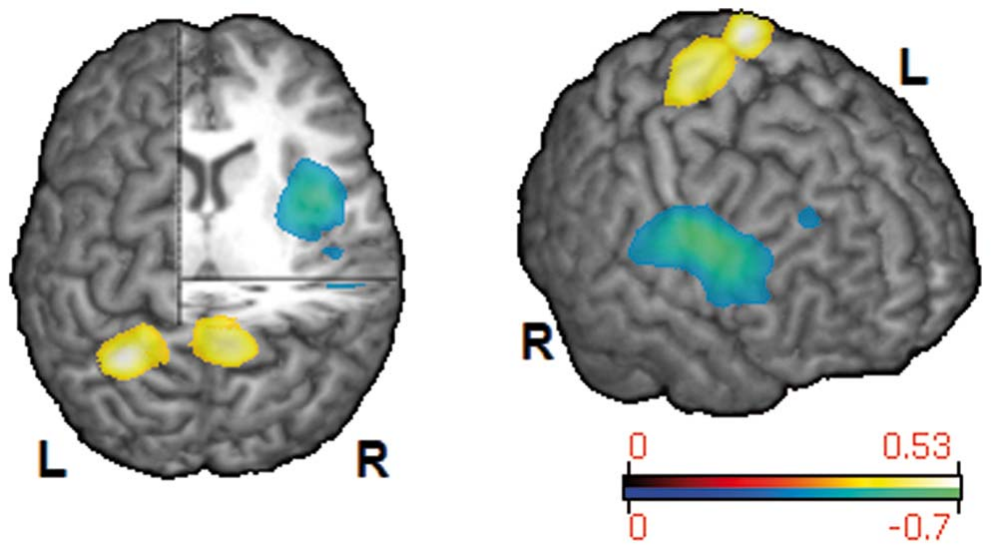
Cortical areas showing a significant difference of swallowing-associated activation between groups (13–30 Hz, p<0.01). The relative increase (blue-green) or decrease (red-yellow) of ERD in patients compared to healthy subjects is color-coded.

In healthy subjects the broadest and strongest event-related desynchronization was found in the beta band (13–30 Hz, figure 2). There was also statistically significant ERD in adjacent frequency bands (alpha, low gamma), which was similarly located but markedly weaker. In dysphagic patients, significant swallow-related cortical activation was exclusively found in the beta frequency range (13–30 Hz, figure 2). The overall strength of ERD in the beta band was comparable between patients and healthy volunteers with similar peak values (−0.5948 and −0.5253, respectively). Healthy subjects displayed bilateral activation of rostro-medial parts of the primary and secondary sensorimotor cortices (table 2), with nearly symmetrical distribution over both hemispheres (LI = 0.0725). In contrast to that, activation in patients with functional dysphagia was centered in caudo-lateral parts of the primary and secondary sensorimotor cortices. Moreover, patients showed prominent lateralization of activation to the right hemisphere (LI = −0.5050), in which the insula, dorsolateral prefrontal cortex (DLPFC) and inferolateral parietal lobe (IPL) were also found to be activated during swallowing. Figure 3 shows areas with statistically significant activation differences between patients and matched volunteers (p<0.01). Patients had focal reduction of ERD in the left medial primary sensory cortex (Brodmann Area (BA) 1–3) and right medial primary sensorimotor cortex and adjacent supplementary motor area (SMA, BA 1–4, 6), whereas a prominent increase of ERD was observed in the right insula, DLPFC, caudolateral motor and premotor cortex (PMC) and IPL (BA 1–4, 6, 9, 13, 40, 43, 44, 45) as compared to healthy subjects.

**Table 2:** Localization of significant swallowing-associated cortical activation per group (13–30 Hz, p<0.05).

Spalte1	Peak location (x, y, z)	Pseudo-t value	Cortical label	BA
**Healthy subjects**				
	l:[−30 −42 69]]	−0.5948	GPrC, GPoC, GFm, LPs,	1–4,6
	r:[ 6 −36 72	−0.5377	GPrC, GPoC, GFm,	1–4,6
**Patients**				
	l:[−45 −24 30]	−0.3237	GPrC, GPoC, GFm, LPi	1–4,6
	r:[ 45 −9 27]	−0.5253	GPrC, GPoC, GFm/i, LPi, Ins	1–4,6,9,13,40,44,45

BA = Brodmann Area, GPrC = Gyrus precentralis, GPoC = Gyrus postcentralis, GFm = Gyrus frontalis medialis, GFi = Gyrus frontalis inferior, LPs = Lobulus parietalis superior, LPi = Lobulus parietalis inferior, Ins = Insula. l = left hemisphere, r = right hemisphere.

## Discussion

In this pilot study we investigated for the first time the cortical swallowing processing in patients with functional dysphagia compared to matched control subjects. According to our findings, functional dysphagia - a condition with assumed normal brain function - seems to be associated with distinctive changes of the swallow-related cortical activation pattern.

The areas that were involved in swallowing in control participants were consistent with previous MEG studies in healthy subjects by our group [10], [11], [17], [22]–[24] corroborating the robustness and reliability of the observed activation pattern in frequency and spacial distribution. As reason for the widespread cortical activation projections to and from the swallowing tract represented in secondary sensorimotor areas were suggested by Hamdy and co-workers [25].

### Cortical activation changes in functional dysphagia patients

In patients increased activation was found in the insula, DLPFC, PMC and IPL of the right hemisphere. In the light of current neuroimaging literature on conversion symptoms as well as functional digestive disorders these regions constitute interfaces between inner perception of body signals, its cognitive appraisal, attentional control and higher order sensorimotor processing. Disbalance and disturbed interaction in this network may result in oropharyngeal hypersensitivity with enhanced self-monitoring leading towards functional symptom perception during otherwise normal deglutition. In the following, the relevance of each brain area, in which activation changes were observed, will be discussed in the context of this hypothesis in more detail.

The insula is a multifunctional region that was found to be activated during swallowing in several studies [22], [26]–[28]. Together with BA 43 and the frontal-parietal operculum (BA 44) it is involved in in the sensory processing of oropharyngeal stimuli like taste, bolus texture and temperature [29]–[32]. The insula is concerned with proprioception and sense of volitional deglutition movements such as sensing awareness of the tongue and other oropharyngeal structures that propel a bolus through the oral cavity [33]. Due to its connections with sensory and motor cortical and subcortical areas it is well suited for multisensory [31] and sensorimotor integration [33], [34] and likely coordinates the temporal sequence of oropharyngeal movements during a swallow [27], [35], thereby participating in the continuous adaption of the motor program to the momentary situation based on afferent sensory information.

Less specific to deglutition, the insula is connected with limbic structures and is said to process the emotional aspects of perception. Particularly the insula and the interconnected DLPFC of the right, non-dominant hemisphere are thought to play a role in interoception, cognitive appraisal and emotional response to unpleasant body signals or visceral pain [36], [37]. For example, patients with irritable bowel syndrome [38] or reflux disease [39] but also healthy volunteers [40], [41] show strong activation in predominantly the right insula and DLPFC to non-painful [39], [41] or painful [38], [40] visceral stimulation. Furthermore, the insula was shown to be involved in the generation of functional somatic syndromes [8].

The DLPFC is especially concerned with self-awareness and attentional control. Due to its connections with limbic, insular and premotor areas it is also involved in sensorimotor integration and thought to subserve higher order guidance of internally-generated action selection [42]–[45]. Internal movement generation, initiation and execution are major functions of the SMA [46]. Its activation was reduced in our patients in the right hemisphere, in which increased DLPFC and insular activation was found. We tentatively interpret this finding as evidence that similar to motor conversion symptoms, the primary motor planning system in our patients is less active whereas structures involved in assigning salience are exceptionally overactive [45]. Because decreased SMA activity is related to impaired voluntary movement initiation, e.g. in Parkinson's Disease [24], [47], [48], it might be speculated that reduced SMA activation could be the reason for a subtle disturbance of movement programming and execution, which might have caused the subjective impression of “difficulty initiating a swallow” or “food getting stuck in the throat” in a setting of enhanced self-monitoring. This notion is supported by a trend towards longer duration of the preparatory oral swallow phase (M0–M1) in psychogenic dysphagia patients (p<0.1).

The above mentioned structures can show positive or negative functional coupling with varying strength of connectivity to the SMA under different circumstances, for example conversion disorder with positive (dystonia, gait abnormalities) or negative (paralysis) motor symptoms [44], [45], [49]. Thus, with the methods applied here, we cannot unravel whether the SMA was suppressed by increased top-down inhibitory influence of these aroused higher-order motor control systems, although our data might suggest this interpretation. The same may be the case in the primary somatosensory cortex, in which activation was bilaterally reduced in our patient population. It is known that the primary sensory cortex reacts more to neutral stimuli whereas the prefrontal cortex shows stronger activation with pleasant or unpleasant percept [50] and the inhibitory influence of the DLPFC on the primary sensory cortex has previously been demonstrated in conversion paralysis [44].

The IPL, that likewise showed increased activation in functional dysphagia patients, is also part of the fronto-parietal attention and self-monitoring network [51]. Tracy et al. (2007) [52] demonstrated the relevance of the right, non-dominant parietal cortex in monitoring inner body signals and focusing attention on one's internal visceral state. With regard to deglutition, the IPL has been suggested to process proprioceptive feedback from the oral cavity and to integrate it with ongoing motor output [53].

Taken together, the observed activation changes in our functional dysphagia patients likely reflect exaggerated activation of a widely distributed vigilance, self-monitoring and salience rating network that interferes with down-stream deglutition sensorimotor control. One may speculate that enhanced self-awareness during swallowing accompanied by negative cognitive appraisal of the sensation leads to emotional arousal and further increases attention towards the putative physical symptom, which finally results in fixed hypersensitivity to oropharyngeal afferent input. Subsequent plastic reorganization of relevant neuronal circuits may then lead to persistent functional dysphagia. This theory of visceral hypersensitivity development produced by interactions of emotional and attentional systems with the sensory-discriminant system [54] has already been proposed for other functional digestive disorders [37]. Psychological factors may constitute a starting point of this unconscious vicious circle, whose outcome is consciously perceived as an organic symptom [55], [56]. However, the observed alterations in cortical activation pattern may either represent pre-existing vulnerability, predisposing these subjects to the development of functional symptoms, or a form of maladaptive cortical plasticity arising from the patient's persistent fixation on his deglutition.

### Clinical implication

Our findings provide a neuropsychophysiological model of functional dysphagia, suggesting that patient management needs a holistic approach to influence central symptom processing. Because functional symptoms are typically more severe when a patient focusses attention on it [55], [57] cognitive behavioral psychotherapy should attempt to reduce emotional factors, arousal and self-awareness that uphold the symptom. Assuming that maladaptive cortical changes partly account for functional dysphagia development, it would be reasonable to block or reverse this abortive plasticity. Spectacular recovery of psychogenic aphonia after a single session of motor cortex transcranial magnetic stimulation was recently reported [58]. Furthermore, the ability of transcranial stimulation techniques to excite or inhibit swallow-related cortical areas has been demonstrated in a variety of studies [23], [59], [60]. Hence, transcranial stimulation in functional dysphagia patients might be an option to restore appropriate network interactions by downregulating emotional insular and attentional prefrontal regions or activating suppressed swallow-related cortical areas.

### Limitations

Our study is limited by the small number of patients that could be recruited, because functional dysphagia is a rare disease. Therefore the interpretation of our findings has to be made with caution and the conclusions drawn – though intriguing – must remain preliminary until our results have been reproduced in a larger patient population. However, it is certainly remarkable that these prominent alterations of the cortical activation pattern were found already in such a small patient group. In the seclusion of the MEG chamber the patients probably were exceptionally mindful of their swallowing, which is known to influence subjective symptom experience as well as cortical activation maps [55], [57]. This fact may have enhanced the neurophysiological alterations in cortical swallowing processing in patients. Moreover, it should be emphasized that statistical analysis was performed with a method that comprises full type 1 error control for both, significant activation per study group and intergroup comparison. Swallowing performance was comparable between both study groups making it unlikely that variety in task performance accounts for the observed activation differences.

## Conclusion

To the best of our knowledge the present study is the first to examine the pattern of cortical swallowing processing in patients with functional dysphagia. Though clearly preliminary, our results are thought-provoking, as they provide a mechanistic explanation for a condition that is generally said to be of psychogenic origin.
